# Trends Following Lumbar Spine Fusion in People Living With Human Immunodeficiency Virus in New York State

**DOI:** 10.7759/cureus.91828

**Published:** 2025-09-08

**Authors:** Bianca A Duah, Mina Botros, Lancelot Benn, Kevin Yoon, Peter Joo, Gabriel Ramirez, Caroline Thirukumaran, Addisu Mesfin

**Affiliations:** 1 Medicine, University of Rochester School of Medicine and Dentistry, Rochester, USA; 2 Orthopaedic Surgery, University of Rochester Medical Center, Rochester, USA; 3 Orthopaedic Surgery, MedStar Washington Hospital Center, Washington, DC, USA; 4 Orthopaedics, Yale New Haven Hospital, New Haven, USA; 5 Orthopaedic Surgery, University of Rochester, Rochester, USA; 6 Orthopaedic Surgery, Northwestern University, Chicago, USA; 7 Orthopaedic Spine Surgery, MedStar Washington Hospital Center, Washington, DC, USA

**Keywords:** complications, human immunodeficiency virus, lumbar fusion surgery, lumbar spine fusion, new york state, outcomes, sparcs, trends

## Abstract

Introduction: The population of individuals living with human immunodeficiency virus (HIV) is aging, resulting in increased rates of degenerative spinal conditions requiring surgical management. Limited literature exists comparing postoperative outcomes of lumbar spine fusion between patients with and without HIV, particularly for patients with CD4 counts higher than 200 (outside of AIDS diagnosis). This study evaluates perioperative complications, length of hospital stay, and discharge outcomes in HIV-positive versus HIV-negative patients following lumbar spine fusion in New York State.

Methods: A retrospective cohort study was performed using the New York Statewide Planning and Research Cooperative System (SPARCS) database, identifying adults undergoing lumbar spine fusion between 2016 and 2021. Patients were stratified by HIV status. Demographics, comorbidities, perioperative complications, mortality, and discharge status were compared using bivariate and multivariable regression analyses. SPARCS provided a composite complication measure was also recorded. Data are presented as n (%), means, and adjusted odds or incidence rate ratios where appropriate.

Results: A total of 75,688 patients underwent lumbar spine fusion, including 526 (0.7%) with HIV and 75,162 (99.3%) without HIV. HIV-positive patients had a mean age of 57 years and were predominantly male (359, 68.3%), compared to the HIV-negative cohort (mean age: 57 years; 37,551, 50.0% male). HIV-positive patients had higher average comorbidities (2.76 vs. 2.04), more frequently experienced non-home discharge (285, 54.2% vs. 31,196, 41.5%), and had a longer mean length of stay (7.0 ± 11.3 days vs. 4.3 ± 5.9 days; p<0.001). The rate of composite index complications was similar (38, 7.2% in HIV-positive vs. 4,790, 6.4% in HIV-negative; p=0.426). The composite score created by SPARCS data encompasses complications such as embolism, renal failure, postoperative abscess, infection, and all-cause hospital mortality. Adjusted analyses confirmed that HIV-positive status was associated with increased length of stay (incidence rate ratio (IRR): 1.28, 95% CI: 1.21-1.36) and higher odds of non-home discharge (OR: 1.58, 95% CI: 1.32-1.89), but not increased complications or 30-day mortality.

Conclusions: HIV-positive patients undergoing lumbar spine fusion experience greater medical complexity and longer hospitalizations and are more frequently discharged to non-home settings than those without HIV. However, the overall rate of perioperative complications and short-term mortality does not differ significantly by HIV status. These findings support careful perioperative planning for HIV-positive individuals undergoing spine surgery to optimize outcomes.

## Introduction

There are approximately 38 million individuals living with human immunodeficiency virus (HIV) infection in the world, with 1.2 million cases active in the United States. Advances in diagnostic efforts and increased access to treatment have delayed progression to acquired immunodeficiency syndrome (AIDS) and subsequently prolonged life expectancy. Consequently, this population is now faced with comorbidities that come with age, such as degenerative lumbar spine disease [1]. Such degenerative spinal conditions are especially prevalent in the United States, affecting up to two-thirds of adults throughout their lifetime [2]. Patients with HIV are prone to developing these conditions, as the disease pathophysiology increases the risk of osteopenia and osteoporosis due to vitamin D deficiency and low body weight. Concurrently, patients with HIV also have low bone mineral density due to alterations in bone metabolism caused by highly active antiretroviral therapy (HAART), HIV viral proteins, and chronic inflammation [3]. From a metabolic standpoint, HIV is associated with a high risk of insulin resistance and type 2 diabetes, dyslipidemia, and metabolic syndrome, which may contribute to increased risk of osteoporosis and fractures [3]. Thus, contributions of the disease process coupled with medications are at a minimum to the earlier age of presentation of HIV patients for elective spinal fusion.

Indications for lumbar spine fusion include mechanical spinal stenosis, spondylolisthesis, fractures, or malignancy [4]. Despite the increase in spinal fusion rates for degenerative disc disease [5,6], the literature is limited in studies reporting potential disparate trends and postoperative outcomes within this population compared to non-HIV-positive patients, regarding lumbar spine fusion. Donnally et al.’s [7] study, published in 2018, investigated one-year outcomes in patients undergoing lumbar fusion and highlighted that, within one year post elective lumbar fusion, HIV-positive patients had an increased risk for respiratory complications, neurologic complications, and higher mortality rates compared with non-HIV-positive patients [7]. This was, however, a nationwide database and may differ in specific regions of the United States - such as New York State.

The study aims to determine if patients with HIV experience disparate postoperative outcomes and complications compared to HIV-negative patients through a retrospective cohort study using patient records from a statewide database, which provides a smaller patient group that may be more homogenous for cultural, environmental, and social risk factors. Mainly, we hope to explore whether outcomes and rates of complications differ or are equivalent between HIV-positive and HIV-negative patients undergoing lumbar fusion. Further, we hope to explore the level of congruence of the findings with the already existing literature.

## Materials and methods

We conducted a retrospective cohort study using the New York Statewide Planning and Research Cooperative System (SPARCS) database from 2016 to 2021 [8]. SPARCS is an all-payer database that compiles patient-level data on diagnoses, treatment services, and inpatient hospital admissions across New York State. This study was approved via expedited review by the institutional review board.

The key predictor was the HIV status of the patient, which was identified using a binary indicator of HIV status obtained as part of the SPARCS dataset. Patients were included if they underwent primary lumbar spine fusion for degenerative disease, identified using International Classification of Diseases, Tenth Revision, Clinical Modification (ICD-10-CM) codes. Due to the nature of ICD and CPT codes, we were unable to differentiate patients with prior lumbar surgery. Patients with degenerative disease were first identified with following ICD-10 codes: M51.06, M51.16, M51.17, M51.26, M51.27, M51.36, M51.37, M51.86, M51.87. They were then filtered for those who had the following ICD-10 lumbar fusion codes: 0SG0070, 0SG0071, 0SG007J, 0SG00A0, 0SG00AJ, 0SG00J0, 0SG00J1, 0SG00JJ, 0SG00K0, 0SG00K1, 0SG00KJ, 0SG0370, 0SG0371, 0SG037J, 0SG03A0, 0SG03AJ, 0SG03J0, 0SG03J1, 0SG03JJ, 0SG03K0, 0SG03K1, 0SG03KJ, 0SG0470, 0SG0471, 0SG047J, 0SG04A0, 0SG04AJ, 0SG04J0, 0SG04J1, 0SG04JJ, 0SG04K0, 0SG04K1, 0SG04KJ, 0SG1070, 0SG1071, 0SG107J, 0SG10A0, 0SG10AJ, 0SG10J0, 0SG10J1, 0SG10JJ, 0SG10K0, 0SG10K1, 0SG10KJ, 0SG1370, 0SG1371, 0SG137J, 0SG13A0, 0SG13AJ, 0SG13J0, 0SG13J1, 0SG13JJ, 0SG13K0, 0SG13K1, 0SG13KJ, 0SG1470, 0SG1471, 0SG147J, 0SG14A0, 0SG14AJ, 0SG14J0, 0SG14J1, 0SG14JJ, 0SG14K0, 0SG14K1, 0SG14KJ, 0SG3070, 0SG3071, 0SG307J, 0SG30A0, 0SG30AJ, 0SG30J0, 0SG30J1, 0SG30JJ, 0SG30K0, 0SG30K1, 0SG30KJ, 0SG3370, 0SG3371, 0SG337J, 0SG33A0, 0SG33AJ, 0SG33J0, 0SG33J1, 0SG33JJ, 0SG33K0, 0SG33K1, 0SG33KJ, 0SG3470, 0SG3471, 0SG347J, 0SG34A0, 0SG34AJ, 0SG34J0, 0SG34J1, 0SG34JJ, 0SG34K0, 0SG34K1, and 0SG34KJ.

Patient-level covariates included HIV status based on the binary indicator provided within SPARCS at the time of surgery. As well as patient demographics, insurance type, admission source, and year of surgery were collected. Race was recorded via patient-level data available through SPARCS. Data were collected for analysis. Age was analyzed as a continuous variable, while insurance status was categorized as private, Medicare, Medicaid, Workers’ Compensation, or other. The Elixhauser AHRQ-Web ICD-10-CM coding algorithm was used to identify 30 pre-existing comorbidities [9]. The HIV ICD code was excluded to avoid confounding.

The primary outcome was the presence or absence of at least one composite index complication within one year of discharge, encompassing cardiac, neurologic, respiratory, gastrointestinal, and oncologic events. Secondary outcomes included length of hospital stay, discharge disposition (home vs. non-home location), and 30-day mortality. Discharge status was categorized as either home or non-home (e.g., skilled nursing, rehabilitation, or hospice facility).

Descriptive statistics were calculated using chi-square tests for categorical variables and Kruskal-Wallis tests for continuous variables to compare HIV-positive and HIV-negative groups. Multivariable analyses were performed using binomial and logistic regression models to assess the relationship between HIV status and outcomes (length of stay, discharge status, complications, and 30-day mortality). Covariates and key predictors are listed above that were used for multivariable regression. Binomial regression was used for length of stay, and logistic regression was used for discharge status, complications, and 30-day mortality. Variable selection was chosen due to the potential confounding effect on results and to normalize patient-to-patient differences. Results with n ≤11 were redacted to maintain patient confidentiality. Propensity matching was performed by age, gender, payor, point of origin, and discharge year to compare HIV-positive and HIV-negative patients. The propensity score was estimated using logistic regression using the covariates mentioned previously. 

This publication is based on data provided by the New York State Department of Health (NYSDOH), but the findings and conclusions are solely those of the authors and do not represent official NYSDOH policy.

## Results

Study population and baseline demographics

A total of 75,688 patients underwent lumbar spine fusion during the study period, comprising 526 (0.7%) individuals with HIV and 75,162 (99.3%) without HIV (Table 1). This value aligns with the published New York prevalence of 742 per 100,000 (or 0.742%). The mean age at surgery was 56.6 years (SD: 10.1) for HIV-positive and 56.9 years (SD: 15.9) for HIV-negative patients. The HIV-positive cohort was predominantly male, with 359 (68.3%) males and 167 (31.8%) females, compared to the HIV-negative cohort, which had a nearly even gender distribution - 37,551 (50.0%) males and 37,608 (50.0%) females. Regarding race, most HIV-negative patients were White (52,975, 70.5%) individuals, while Black patients comprised 8,032 (10.7%) in the HIV-negative and 20 (3.8%) in the HIV-positive cohort. Elective admissions accounted for 384 (73.0%) HIV-positive and 62,321 (82.9%) HIV-negative patients. The average comorbidity burden, as measured by the Elixhauser index, was higher among HIV-positive patients, with a mean of 2.76 (SD: 0.01), compared to 2.04 (SD: 1.68) in HIV-negative individuals.

**Table 1:** Baseline characteristics HIV = Human immunodeficiency virus Demographic breakdown of all patients within the New York Statewide Planning and Research Cooperative System (SPARCS) database who underwent lumbar fusion, separated by HIV status. Provided to show a general breakdown of the population found within the SPARCS database and to display variables that will be used in eventual propensity matching further in the paper. Although low rates of White individuals in HIV-positive patients, this aligns with reported data that the majority of HIV-positive patients are non-white in New York State. Thus, generalizability still stands. Significant p-value is considered p < 0.05, and all descriptive statistics were calculated using the chi-square tests for categorical variables and Kruskal-Wallis tests for continuous variables between HIV-positive and HIV-negative groups.

Characteristic	HIV-Negative (n=75,162)	HIV-Positive (n=526)	p-value
Age, mean (SD)	56.9 (15.9)	56.6 (10.1)	0.003
Male, n (%)	37,551 (50.0)	359 (68.3)	<0.001
White, n (%)	52,975 (70.5)	21 (4.0)	<0.001
Black, n (%)	8,032 (10.7)	20 (3.8)	<0.001
Other Race, n (%)	12,071 (16.1)	13 (2.5)	0.002
Elective Admission, n (%)	62,321 (82.9)	384 (73.0)	<0.001
Medicaid, n (%)	5,412 (7.2)	72 (13.7)	<0.001
Medicare, n (%)	24,615 (32.8)	176 (33.5)	0.791
Admission from Health Facility, n (%)	14,680 (19.5)	150 (28.5)	<0.001
Mean Elixhauser Index (SD)	2.04 (1.68)	2.76 (2.01)	N/A
Non-home Discharge, n (%)	31,196 (41.5)	285 (54.2)	<0.001

Comorbidity profiles

HIV-positive patients demonstrated a greater prevalence of certain comorbidities. Renal failure was present in 44 (8.4%) HIV-positive patients versus 3,610 (4.8%) HIV-negative patients (p=0.017). Weight loss was documented in 26 (4.9%) HIV-positive patients and 847 (1.1%) HIV-negative patients (p<0.001). Fluid and electrolyte disorders were more common among those with HIV (74, 14.1% vs. 7,797, 10.4%; p=0.006), as was complicated hypertension (44, 8.4% vs. 4,339, 5.8%; p=0.011) (Table 2). Other comorbidities, including uncomplicated hypertension, chronic pulmonary disease, and diabetes, did not differ significantly between the two groups.

**Table 2:** Comorbidity prevalence (matched cohort) HIV = Human immunodeficiency virus The table shows the breakdown of common comorbidities found among patients who underwent lumbar fusion within the New York Statewide Planning and Research Cooperative System (SPARCS) database. The SPARCS database has the rates of these comorbidities available with the extracted patient data. The rates of comorbidities were then separated into patients who underwent lumbar fusion, who were either HIV-positive or HIV-negative. Comorbidities were shown to indicate the varying factors that could play a role outside of HIV alone. P-value < 0.05 was significant. P-value is based on the comparison of rates of comorbidities between two groups separated by HIV status. P-value was calculated using the chi-square test between HIV-positive and HIV-negative groups.

Comorbidity	HIV-Negative n (%)	HIV-Positive n (%)	p-value
Renal Failure	3,610 (4.8)	44 (8.4)	0.017
Weight Loss	847 (1.1)	26 (4.9)	<0.001
Fluid/Electrolyte Disorders	7,797 (10.4)	74 (14.1)	0.006
Hypertension, Complicated	4,339 (5.8)	44 (8.4)	0.011
Chronic Pulmonary Disease	13,200 (17.6)	135 (25.7)	<0.001
Liver Disease	1,168 (1.6)	42 (8.0)	<0.001
Drug Abuse	1,811 (2.4)	69 (13.1)	<0.001

Length of stay

The median length of stay for HIV-positive patients was 4.0 days (IQR: 2.0-7.0) versus 3.0 days (IQR: 2.0-5.0) for HIV-negative patients. After adjusting for comorbidities, HIV-positive status was associated with a 28% longer length of stay (incidence rate ratio (IRR): 1.28, 95% CI: 1.21-1.36; p<0.001) compared to HIV-negative patients (Table 3). Propensity-matched analysis showed that HIV-positive patients had a 30% longer length of stay (IRR: 1.30, 95% CI: 1.17-1.43; p<0.001).

**Table 3:** Adjusted outcomes - regression analysis IRR = Incidence rate ratio; OR = Odds ratio; CI = Confidence interval; LOS = Length of stay; HIV = Human immunodeficiency virus The table shows four main surgical outcomes analyzed within the study, which include length of stay, discharge location (home versus non-home), complications, and 30-day mortality rate. Non-home discharge locations included long-term care facilities, nursing facilities, and short-term rehabilitation facilities. Complications are entered within the New York Statewide Planning and Research Cooperative System (SPARCS) database via International Classification of Diseases (ICD) 9/10 codes and are available with initial data extraction. P-value of < 0.05 was considered significant. IRR, OR, and p-values were calculated using binomial and logistic regression models to assess the relationship between HIV status and outcomes listed above. Propensity matching was performed by age, gender, payor, point of origin, and discharge year to compare HIV-positive and HIV-negative patients.

Variable	Estimate Type	Estimate	95% CI	p-value
HIV Positive (vs Negative)	IRR (LOS)	1.28	1.21–1.36	<0.001
HIV Positive (vs Negative)	OR (Non-home Discharge)	1.58	1.32–1.89	<0.001
HIV Positive (vs Negative)	OR (Complication)	0.97	0.69–1.36	0.86
HIV Positive (vs Negative)	OR (30d Mortality)	2.13	0.51–8.91	0.30

Discharge disposition

**Figure 1:**
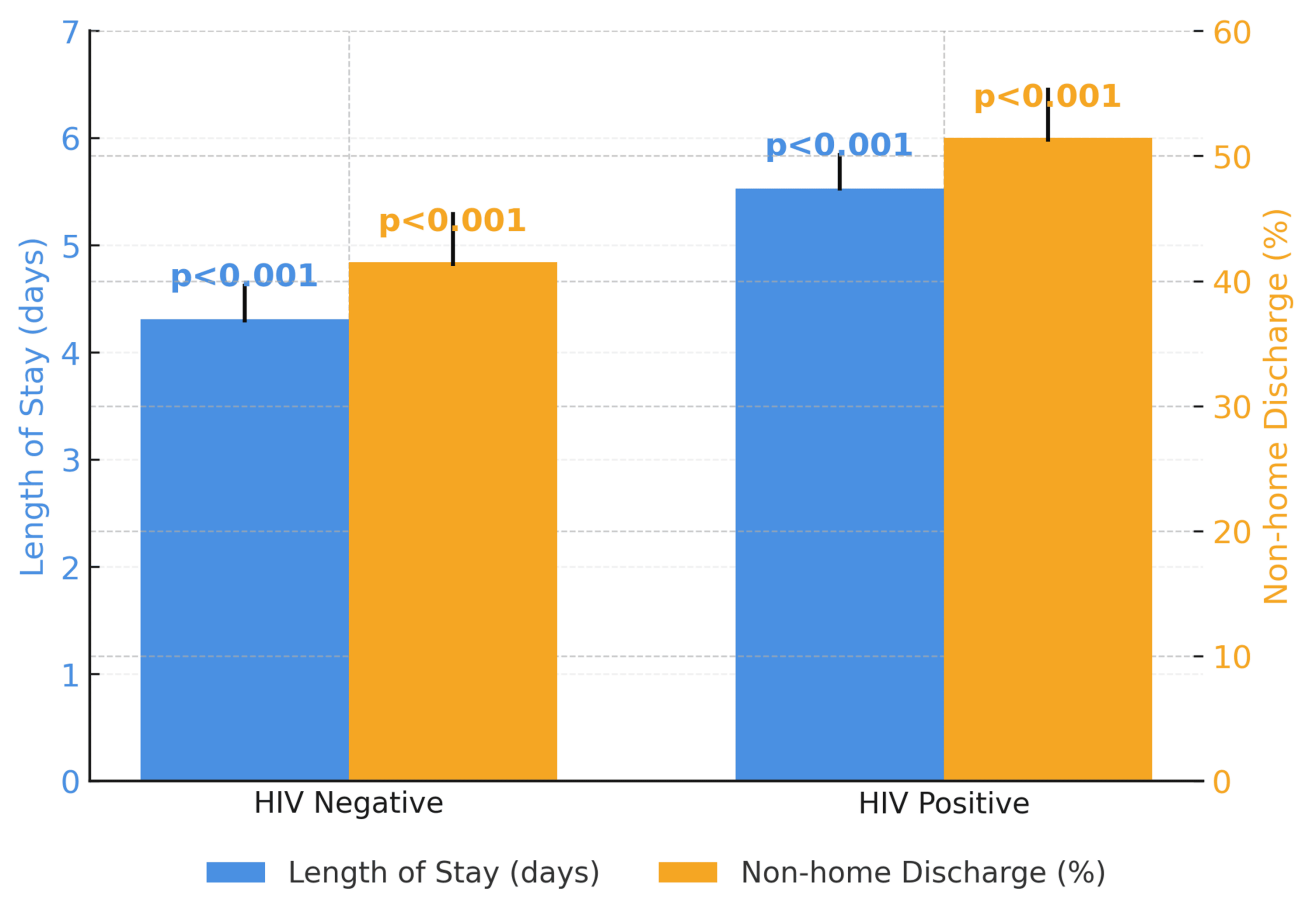
Adjusted mean length of hospital stay and probability of non-home discharge by HIV status HIV = Human immunodeficiency virus Error bars represent 95% confidence intervals. P-values from the adjusted regression models are shown above the bars, presented with the same data as Table 4 (adjusted means after propensity matching) but shown in a bar-graph form for ease of visual comparison. P-value < 0.05 is considered significant.

A higher proportion of HIV-positive patients were discharged to a non-home setting following surgery, with 285 (54.2%) discharged to a facility other than home compared to 31,196 (41.5%) in the HIV-negative group (p<0.001). In adjusted regression models, HIV-positive patients had higher odds of non-home discharge (OR: 1.58, 95% CI: 1.32-1.89; p<0.001) compared to HIV-negative patients (Table 3). In the matched cohort analysis, HIV-positive status remained associated with increased odds of non-home discharge (OR: 1.63, 95% CI: 1.25-2.11; p<0.001) (Table 4, Figure 1).

**Table 4:** Adjusted mean outcomes HIV = Human immunodeficiency virus; CI = Confidence interval The table presents data of adjusted mean outcomes, which were calculated after propensity matching was performed by age, gender, payor, point of origin, and discharge year between patients who were HIV-positive and HIV-negative. The adjusted means were then analyzed with a Kruskal-Wallis test to calculate the p-value. P-value < 0.05 was considered significant.

Outcome	HIV-Negative	HIV-Positive	Mean Difference (95% CI)	p-value
Mean Length of Stay (days)	4.31 (4.28–4.33)	5.53 (5.20–5.86)	1.22 (0.89–1.55)	<0.001
Non-home Discharge Probability (%)	41.52 (41.19–41.85)	51.47 (47.50–55.45)	9.95 (5.96–13.94)	<0.001
Complication Probability (%)	6.38 (6.21–6.55)	6.21 (4.29–8.12)	–0.18 (–2.10–1.75)	0.86
30-day Mortality Probability (%)	0.32 (0.27–0.38)	0.67 (–0.24–1.58)	0.35 (–0.57–1.26)	0.30

Composite index complications

The rate of composite index complications during the index hospitalization was 38 (7.2%) for HIV-positive patients and 4,790 (6.4%) for HIV-negative patients (p=0.426). After adjustment, HIV-positive status was not significantly associated with the risk of complications (OR: 0.97, 95% CI: 0.69-1.36; p=0.86). The adjusted mean probability of complications was 6.2% for HIV-positive and 6.4% for HIV-negative patients (Table 5).

**Table 5:** Propensity-matched outcomes IRR = Incidence rate ratio; OR = Odds ratio; CI = Confidence interval; LOS = Length of stay; HIV = Human immunodeficiency virus The table presents the results of a multivariable analyses using binomial and logistic regression to assess the relationship between HIV status and length of stay and non-home discharge after patient data had been propensity matched by age, gender, payor, point of origin, and discharge year to compare HIV-positive versus HIV-negative patients. P-value < 0.05 was considered significant. P-value was found using binomial and logistic regression models to assess for relationship between HIV status and length of stay and discharge status.

Variable	IRR/OR	95% CI	p-value
Length of Stay	1.30	1.17–1.43	<0.001
Non-home Discharge	1.63	1.25–2.11	<0.00

Thirty-day mortality

Thirty-day mortality was rare in both cohorts. Among HIV-positive patients, the event rate was below the reporting threshold (n≤11). Among HIV-negative patients, 124 (0.16%) deaths were recorded. In adjusted analysis, HIV-positive status was not significantly associated with 30-day mortality (OR: 2.13, 95% CI: 0.51-8.91; p=0.30) (Table 2). The adjusted mean probability of 30-day mortality was 0.67% for HIV-positive and 0.32% for HIV-negative patients (Table 5).

## Discussion

In the past 20 years, there has been a significant increase in spinal surgery amongst HIV-positive patients as per a systematic review by Farias et al., with spine surgeries increasing at 0.094 incidences per 100,000 in 2000 and 0.303 in 2009 [10]. However, the literature is limited in the examination of the impact of HIV status on trends and outcomes following lumbar spine fusion. Our study aimed to add to the current literature regarding potential complications, mortality, postoperative discharge status, length of stay, and mortality amongst patients with HIV undergoing lumbar spine fusion.

Our findings highlight that the percentage of HIV-negative patients remained consistently high and stable, indicating a steady number of procedures among these patients. There was a notable increase in the percentage of HIV-positive patients over the years, suggesting an increasing trend in lumbar spine fusion procedures among this group (Table 1), similar to the findings of Farias et al. [10].

Demographically, our study differed were similar as previous studies. A retrospective study by Lovy et al. [11] noted that, between 2002 and 201,1, among patients undergoing cervical spine surgery, patients with HIV were significantly younger than patients without HIV (48 years vs. 53.4 years; p<0.001) and were more likely to be male. Further, a significantly greater number of HIV-positive patients were Black individuals (27.3% vs. 62.4%) and were more likely to have Medicare and Medicaid. Shah et al. evaluated a propensity-matched analysis of 570 patients who underwent two- to three-level lumbar fusion between HIV-positive and HIV-negative patients [4]. They found that there was statistically insignificant difference observed between patients with HIV and without in age (49.8 vs. 49.7 years; p=0.903), sex (72.3% vs. 71.6% of males; p=0.852), race (0.395), insurance status (0.875), or mean length of stay (4.0 vs. 3.8 days, respectively; p=0.585) [4]. Potentially, variation may be due to the geographic location of studies and representative data.

In our analysis, when accounting for the pre-existing comorbidities that predominated our HIV-positive group, only renal failure and weight loss were statistically significant. Compared with past literature regarding comorbidities, bivariate analysis highlighted that HIV patients had significantly greater odds of having chronic pulmonary disease (OR: 1.76), liver disease (OR: 11.28), coagulopathy (OR: 3.09), and drug abuse (OR: 10.08). Of note, among both HIV-positive and HIV-negative groups, rates of congestive heart failure and diabetes were similar, while the odds of obesity were significantly reduced in HIV-positive patients (OR: 0.58) [11]. To note, there was consideration to group comorbidities by organ system - which may be helpful, especially in this patient population, where multiple areas of the body can be affected.

While we were unable to identify specific complications that occurred with our HIV-positive cohort compared to their HIV-negative cohort, we did identify that HIV-positive patients had a slightly lower rate of index stay complications. Previously, Lovey et al. [11] found that, postoperatively, the most common complication was postoperative hemorrhage, followed by genitourinary concerns. The findings of the current study align with those of Varshneya et al. [2], who found that HIV-positive patients were more likely to experience any postoperative complications (OR: 1.7, 95% CI: 1.2-2.3). Studies that have explored one-year outcomes in patients undergoing lumbar fusion show that HIV-positive patients have an increased risk for respiratory complications (OR: 5.43, 95% CI: 3.40-8.67; p<0.001), wound complications (OR: 2.60; 95% CI: 1.37-4.96; p=0.004), neurologic complications (OR: 1.96; 95% CI: 1.04-3.73; p=0.039), and higher mortality rates (OR: 39.1; 95% CI: 14.99-106.30; p<0.001) compared with non-HIV patients [1,10,12]. Similarly, Shah et al. [13] found that patients with HIV had higher rates of respiratory complications compared to non-HIV patients (4.3% vs. 0.4%; p<0.033). However, the overall rate of complications was similar amongst their HIV vs. non-HIV cohort, a finding like our overall cohort.

Our findings add to the literature demonstrating that, while both the treatment of HIV and disease process itself increase the risk of perioperative complications, which might translate into longer lengths of stay and discharge to non-home postoperative care, our HIV-positive group did not have statistically significant differences in outcomes regarding index stay complications and 30-day mortality compared to their HIV-negative counterparts. This aligns with prior findings where HIV-positive patients undergoing spinal surgery were found to have higher rates of 30-day readmission (11.1% vs. 2.2%; p=0.04), one-year pseudoarthrosis (17.8% vs. 4.4%; p=0.02), and one-year infectious complication rate (13.3% vs. 3.3%; p=0.06) [8].

The relevance of our study is its contribution to preoperative planning and postoperative management of HIV-positive individuals undergoing lumbar spine surgery. A known side effect of HAART is low bone mineral density [14,15], and this translates to an elevated risk of postoperative complications that may require revision surgery - including pedicle screw loosening, nonunion, proximal junctional kyphosis, and adjacent segment fractures [13]. Additionally, people living with HIV have previously shown an increased risk of surgical site infections (SSI) after orthopedic procedures (OR: 1.4; 95% CI: 0.5-3.8) as per Harrison et al. and Kigera et al. [16,17]. However, these findings could not be explored in our analysis due to the limitations of the database.

Limitations

This study has several limitations inherent to the use of a large administrative database. First, the SPARCS dataset does not provide detailed clinical information, such as CD4 count, HIV viral load, antiretroviral therapy status, or AIDS diagnosis, which may influence surgical outcomes. As a result, we could not assess the impact of HIV disease severity or treatment on perioperative risk. Because of the sensitive nature of HIV status, outcomes with sample sizes of 11 or fewer individuals were not reported, potentially limiting the analysis of rare complications. Coding errors and omissions in ICD-10 data may result in misclassification of comorbidities or postoperative events. Additionally, variables such as socioeconomic status, zip code, operative details, and post-discharge outcomes beyond 30 days were not available. Despite multivariable adjustment and propensity matching, the possibility of residual confounding remains. Future studies incorporating more granular clinical and demographic data are needed to further characterize risks and optimize care for HIV-positive patients undergoing lumbar spine fusion.

## Conclusions

Despite the prevalence of HIV, the limited literature regarding outcomes and complications of lumbar spine fusion among HIV-positive patients continues to highlight the paucity of studies regarding the subject. Our study serves as one of the largest studies examining the nuances (or lack thereof) of HIV positivity in the setting of orthopedic procedures. Our study found that HIV-positive status did not significantly affect short-term outcomes (e.g., 30 days) compared to the control group, but did result in a longer mean of stay (seven days vs. four days). Ultimately, our findings suggest that HIV-positive patients are at risk of potential complications and adverse outcomes. Thus, before proceeding with surgical intervention in HIV-positive individuals, surgeons must carefully consider each patient’s specific diagnosis, comorbidities, and risk factors. Surgeons may benefit my risk-stratifying patients by these measures, and by CD4-level, to help guide management, as well as prepare for potential perioperative and postoperative complications.

## References

[1] King JT Jr, Perkal MF, Rosenthal RA (2015). Thirty-day postoperative mortality among individuals with HIV infection receiving antiretroviral therapy and procedure-matched, uninfected comparators. JAMA Surg.

[2] Varshneya K, Wadhwa H, Ho AL (2022). Surgical outcomes of human immunodeficiency virus-positive patients undergoing lumbar degenerative surgery. Clin Spine Surg.

[3] Ahmed M, Mital D, Abubaker NE, Panourgia M, Owles H, Papadaki I, Ahmed MH (2023). Bone health in people living with HIV/AIDS: an update of where we are and potential future strategies. Microorganisms.

[4] Shah NV, Lettieri MJ, Gedailovich S (2023). The impact of asymptomatic human immunodeficiency virus-positive disease status on inpatient complications following spine surgery: a propensity score-matched analysis. J Clin Med.

[5] Best MJ, Buller LT, Eismont FJ (2015). National trends in ambulatory surgery for intervertebral disc disorders and spinal stenosis: a 12-year analysis of the national surveys of ambulatory surgery. Spine (Phila Pa 1976).

[6] Yoshihara H, Yoneoka D (2014). National trends and in-hospital outcomes in HIV-positive patients undergoing spinal fusion. Spine (Phila Pa 1976).

[7] Donnally CJ 3rd, Kalakoti P, Buskard AN (2018). Inpatient outcomes after elective lumbar spinal fusion for patients with human immunodeficiency virus in the absence of acquired immunodeficiency syndrome. World Neurosurg.

[8] Statewide Planning and Research Cooperative System (SPARCS) -Department of Health. Statewide Planning and Research Cooperative System. (2022 (2025). Statewide Planning and Research Cooperative System (SPARCS). August). Retrieved December 19, 2022, from.

[9] Elixhauser A, Steiner C, Harris DR, Coffey RM (1998). Comorbidity measures for use with administrative data. Med Care.

[10] Farias FA, Dagostini CM, Falavigna A (2021). HIV and surgery for degenerative spine disease: a systematic review. J Neurol Surg A Cent Eur Neurosurg.

[11] Lovy AJ, Guzman JZ, Skovrlj B, Cho SK, Hecht AC, Qureshi SA (2015). Prevalence, comorbidities, and risk of perioperative complications in human immunodeficiency virus-positive patients undergoing cervical spine surgery. Spine (Phila Pa 1976).

[12] Ifarraguerri AM, Malyavko A, Stoll WT, Patel S, Thakkar S (2021). Impact of human immunodeficiency virus on 2-year revision rates following lumbar fusion for degenerative spinal conditions: a retrospective cohort study. J Spine Surg.

[13] Shah NV, Lettieri MJ, Scheer R, Sedaghatpour D, Ford B, Diebo BG, Paulino CB (2020). Does asymptomatic human immunodeficiency virus (AHIV)-positive status in patients undergoing spinal fusion for degenerative disc disease (DDD) increase risk for adverse postoperative outcomes?. Spine J.

[14] Pietrzak JR, Maharaj Z, Mokete L, Sikhauli N (2020). Human immunodeficiency virus in total hip arthroplasty. EFORT Open Rev.

[15] Pretell-Mazzini J, Subhawong T, Hernandez VH, Campo R (2016). HIV and orthopaedics: musculoskeletal manifestations and outcomes. J Bone Joint Surg Am.

[16] Harrison WJ, Lewis CP, Lavy CB (2002). Wound healing after implant surgery in HIV-positive patients. J Bone Joint Surg Br.

[17] Kigera JW, Straetemans M, Vuhaka SK, Nagel IM, Naddumba EK, Boer K (2012). Is there an increased risk of post-operative surgical site infection after orthopaedic surgery in HIV patients? A systematic review and meta-analysis. PLoS One.

